# A novel function for the presenilin family member *spe-4*: inhibition of spermatid activation in *Caenorhabditis elegans*.

**DOI:** 10.1186/1471-213X-8-44

**Published:** 2008-04-22

**Authors:** Ryoko Gosney, Wei-Siang Liau, Craig W LaMunyon

**Affiliations:** 1 Department of Biological Science, California State Polytechnic University, Pomona, CA, USA

## Abstract

**Background:**

Sperm cells must regulate the timing and location of activation to maximize the likelihood of fertilization. Sperm from most species, including the nematode *Caenorhabditis elegans*, activate upon encountering an external signal. Activation for *C. elegans *sperm occurs as spermatids undergo spermiogenesis, a profound cellular reorganization that produces a pseudopod. Spermiogenesis is initiated by an activation signal that is transduced through a series of gene products. It is now clear that an inhibitory pathway also operates in spermatids, preventing their premature progression to spermatozoa and resulting in fine-scale control over the timing of activation. Here, we describe the involvement of a newly assigned member of the inhibitory pathway: *spe-4*, a homolog of the human presenilin gene PS1. The *spe-4(hc196) *allele investigated here was isolated as a suppressor of sterility of mutations in the spermiogenesis signal transduction gene *spe-27*.

**Results:**

Through mapping, complementation tests, DNA sequencing, and transformation rescue, we determined that allele *hc196 *is a mutation in the *spe-4 *gene. Our data show that *spe-4(hc196) *is a bypass suppressor that eliminates the need for the spermiogenesis signal transduction. On its own, *spe-4(hc196) *has a recessive, temperature sensitive spermatogenesis-defective phenotype, with mutants exhibiting (i) defective spermatocytes, (ii) defective spermatids, (iii) premature spermatid activation, and (iv) spermatozoa defective in fertilization, in addition to a small number of functional sperm which appear normal microscopically.

**Conclusion:**

A fraction of the sperm from *spe-4(hc196) *mutant males progress directly to functional spermatozoa without the need for an activation signal, suggesting that *spe-4 *plays a role in preventing spermatid activation. Another fraction of spermatozoa from *spe-4(hc196) *mutants are defective in fertilization. Therefore, prematurely activated spermatozoa may have several defects: we show that they may be defective in fertilization, and earlier work showed that they obstruct sperm transfer from males at mating. *hc196 *is a hypomorphic allele of *spe-4*, and its newly-discovered role inhibiting spermiogenesis may involve known proteolytic and/or calcium regulatory aspects of presenilin function, or it may involve yet-to-be discovered functions.

## Background

Sperm cells can be considered as semi-autonomous organisms having their own unique genomes and living lives that are, in part, independent of the organisms that produced them. Sperm are exceedingly variable across animal taxa in terms of morphology, motility, and lifespan. One seemingly invariant feature is that a sperm cell's active life must initiate with precise timing. Precocious activation may result in premature consumption of stored energy or interfere with efficient fertilization. Belated activation may allow competing sperm to gain the advantage in the race to fertilize the ova. In many cases, activation coincides with sperm release from the male. For example, in sea urchins, a longstanding model for sperm function, Na^+ ^enters the sperm when they are released into seawater. A simultaneous pH spike stimulates motility and respiration through a signal transduction pathway that includes spikes in Ca^2+^, cAMP, and cAMP dependent kinases. The result is the activation of dynein ATPase in the flagellum and increased mitochondrial activity [1-3]. The sudden ionic flux that accompanies release into seawater is the cue that initiates sperm motility in sea urchins.

In internal fertilizing species, the sperm also require a cue to initiate activity. The cue is often a signaling molecule. In many mammals, bicarbonate serves as the cue, inducing a Ca^2+ ^influx, cAMP accumulation, cAMP dependent protein kinase A activity, and increased motility and respiration [4-7]. Thus, even though sea urchins and mammals are distantly related and the signals for sperm activation are different, some of the cellular processes involved in sperm activation are similar.

Here, we are concerned with the cellular mechanisms that regulate sperm activation in the nematode *Caenorhabditis elegans*. Like all nematode sperm, those from *C. elegans *are amoeboid, and attainment of activity coincides with the developmental transition from spermatid to spermatozoon (= spermiogenesis). In hermaphrodites, spermiogenesis occurs as the spermatids enter the sperm storage chamber, or spermatheca. In males, spermiogenesis occurs during copulation as the spermatids encounter seminal fluid [8]. In both cases, the timing of spermiogenesis is critical. For instance, belated activation may result in the spermatids being swept from the reproductive tract by passing eggs [9], whereas prematurely activated spermatozoa within the male reproductive tract impede sperm transfer [10]. The signaling molecule that initiates spermiogenesis in *C. elegans *is unknown, but in a manner similar to the species discussed above, sperm activation in *C. elegans *appears to involve the influx of cations [11], a brief elevation in pH [12], and the release of intracellular Ca^2+ ^[8,13,14].

The control of *C. elegans *spermiogenesis involves a number of gene products. The signal to activate is transduced through a pathway that includes the genes *spe-8*, *spe-12 *[15,16], *spe-19 *[17], *spe-27 *[18], and *spe-29 *[19]. Mutations in these genes result in arrest at the spermatid stage in hermaphrodite produced self-sperm. Curiously, mutant males are fertile, and the arrested hermaphrodite produced self-sperm can be "rescued" when exposed to seminal fluid from males, which, along with other data, suggests that this "*spe-8" *group of genes amplifies the activation signal for hermaphrodite self sperm [8,16]. *In vitro*, the arrest associated with mutations in the *spe-8 *group genes can be overcome by inducing one of the events that occurs downstream of the signal transduction pathway: elevation of pH. This is accomplished by treating the cells with the weak base triethanolamine [12]. Thus, *C. elegans *sperm possess a complex spermiogenesis pathway that ensures the timely activation of spermatids.

It is now clear that spermatid activation in *C. elegans *also involves negative regulation. The propensity to undergo spermiogenesis is so great that an inhibitory pathway is required to prevent premature progression through this important developmental transition. This inhibitory pathway includes *spe-6*. A large screen for mutations that suppress the sterile phenotype of *spe-27(it132ts) *identified a number of mutations in *spe-6*, which result in premature spermatid activation and in suppression of mutations of the *spe-8 *group genes [20]. SPE-6 inhibits spermiogenesis until the activation signal is transduced through the *spe-8 *group gene products. SPE-6 also functions earlier in spermatogenesis in the packaging of Major Sperm Protein (MSP) into the fibrous body-membranous organelles (FB-MOs), which are unique to the sperm cells [21]. Here, we report on the inhibitory function of SPE-4 on spermatid activation. *spe-4*, a homolog of the human presenilin gene PS1, had been known for its role in MSP packaging in the FB-MOs early in spermatogenesis [21-23]. Our investigation focuses on a hypomorphic *spe-4 *allele isolated in the same suppressor screen that produced the *spe-6 *suppressor alleles. Like the *spe-6 *suppressor alleles, *spe-4(hc196) *suppresses mutations in the *spe-8 *group genes and results in premature spermatid activation.

## Results and Discussion

### Genetic mapping places *hc196 *right of center on chromosome I

**Figure 1:**
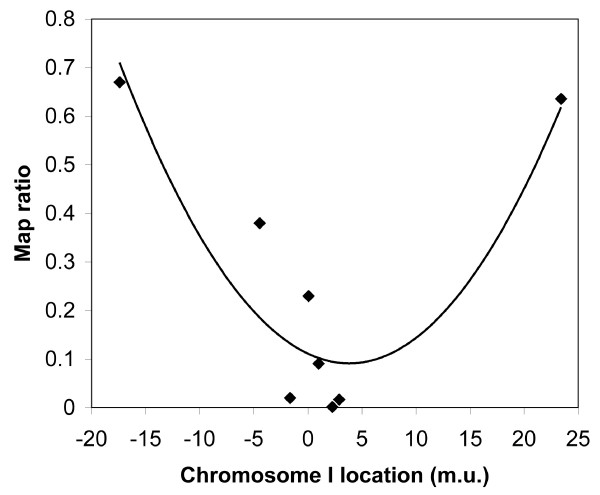
**Single nucleotide polymorphism mapping results**. The map ratio represents the ratio of N2 to CB4658 DNA from the bulk isolated DNA from mutant and wild-type worms. A map ratio near one indicates no linkage, whereas map ratios near zero are found for loci nearby the mapped gene [24]. The curve is the best-fit second order equation: y = 0.0014 × 2 - 0.0105x + 0.1111.

We initially mapped *hc196 *to chromosome I using single nucleotide polymorphisms from the polymorphic Hawaiian strain CB4856. Linkage to chromosome I was unequivocal (map ratio to SNP on chromosome: I – 0.00, II – 1.01, III – 1.63, V – 1.58; ratios near zero indicate linkage [24]). We then undertook mapping with SNP loci across chromosome I. The calculated map ratios for each of the SNP loci analyzed are shown in Fig. 1. The second order equation for the best fit curve (y = 0.0014 × 2 - 0.0105x + 0.1111) has a minimum at 3.75, which is a rough estimate of the locus. Based upon the fact that the SNP loci at 2.26 (pkP1117) and 2.88 (pkP1119) showed almost no recombination with the Hawaiian chromosomes, we chose to conduct a three factor mapping cross with markers that flank this region: *dpy-5 *and *unc-29*, located at 0.00 and 3.32, respectively. The cross was performed in a *spe-27 *background, so the *hc196 *mutant phenotype was fertility. We recovered 34 recombinant F2 progeny: 19 recombination events occurred between *dpy-5 *and *hc196 *(5 fertile *dpy *and 14 sterile *unc*), and 15 recombination events occurred between *hc196 *and *unc-29 *(14 sterile *dpy *and 1 fertile *unc*). Based upon these data, *hc196 *resides at 1.9 m.u. on chromosome I (19/34 × 3.32).

### *hc196 *is an allele of *spe-4*

Due to its proximity to *spe-4 *at 2.04 on chromosome I, we decided to perform a complementation test to determine if *hc196 *is an allele of *spe-4*. Worms with the *hc196/spe-4(hc78) *genotype were sterile (Table 1), indicating that *hc196 *is an allele of *spe-4*. There are six other alleles of *spe-4*, and all are sterile (only *eb27*, a nonsense mutation, produces one offspring per 100 mutant worms [22]). The fact that *hc196 *homozygotes are partially fertile suggests that the allele is hypomorphic and that these worms have some SPE-4 function. Interestingly, the trans-heterozygote, composed of a hypomorphic allele over a null allele (*hc196/hc78*), was sterile (Table 1). Apparently, SPE-4 activity from only a single hypomorphic allele is too little to allow functional sperm production.

**Table 1:** Results of a complementation test between *hc196 *and *spe-4(hc78)*.

**Hermaphrodite Genotype**	**Brood Size ± SEM (N)**
*hc196 *+*/spe-4(hc78) unc-15; spe-27/+*	0.6 ± 0.3 (62)
*spe-4(hc78) unc-15*	0 ± 0 (15)
*hc196*	16.6 ± 1.8 (27)
*spe-4(hc78) unc-15/++*	95.3 ± 5.7 (12)
*hc196/*+	94.8 ± 14.1 (9)
*spe-27/+*	199.3 ± 11.8 (12)

Worms were reared at 25°C.

The identity of *hc196 *as an allele of *spe-4 *was supported by transformation rescue experiments. N2 worms transformed with *myo-2::*GFP and a PCR product containing wild-type *spe-4 *and its flanking DNA (*zqEx1 [myo-2::GFP spe-4(+)]*; see Methods) were mated to *dpy-5 hc196; spe-27 unc-22 *hermaphrodites. We scored the fertility of F2 worms expressing GFP (to select for the transgenes) and a dumpy phenotype (to select for *hc196 *homozygotes) but lacking an uncoordinated phenotype (to avoid the *spe-27 *sterile phenotype). The dumpy transformants had significantly larger brood sizes than did non-transgenic dumpy animals (Table 2; *t *= -5.38, *P *< 0.001), showing that the wild-type *spe-4 *transgene rescued the *hc196 *fertility defect.

**Table 2:** Brood size of worms in transformation rescue experiments.

**Hermaphrodite Genotype**	**Brood Size ± SEM (N)**
*dpy-5 hc196; spe-27 unc-22*	8.8 ± 1.0 (9)
*dpy-5 hc196/++; spe-27 unc-22/++*	63.4 ± 6.1 (7)
*dpy-5 hc196*	14.9 ± 1.0 (11)
Transgenic worms*	53.5 ± 5.7 (15)

* These were *dpy-5 hc196; spe-27 unc-22/++ *or *dpy-5 hc196; ++ *worms transformed with the transgene *zqEx1 [myo-2::GFP spe-4(+)]*. Worms were reared at 25°C.

**Figure 2:**
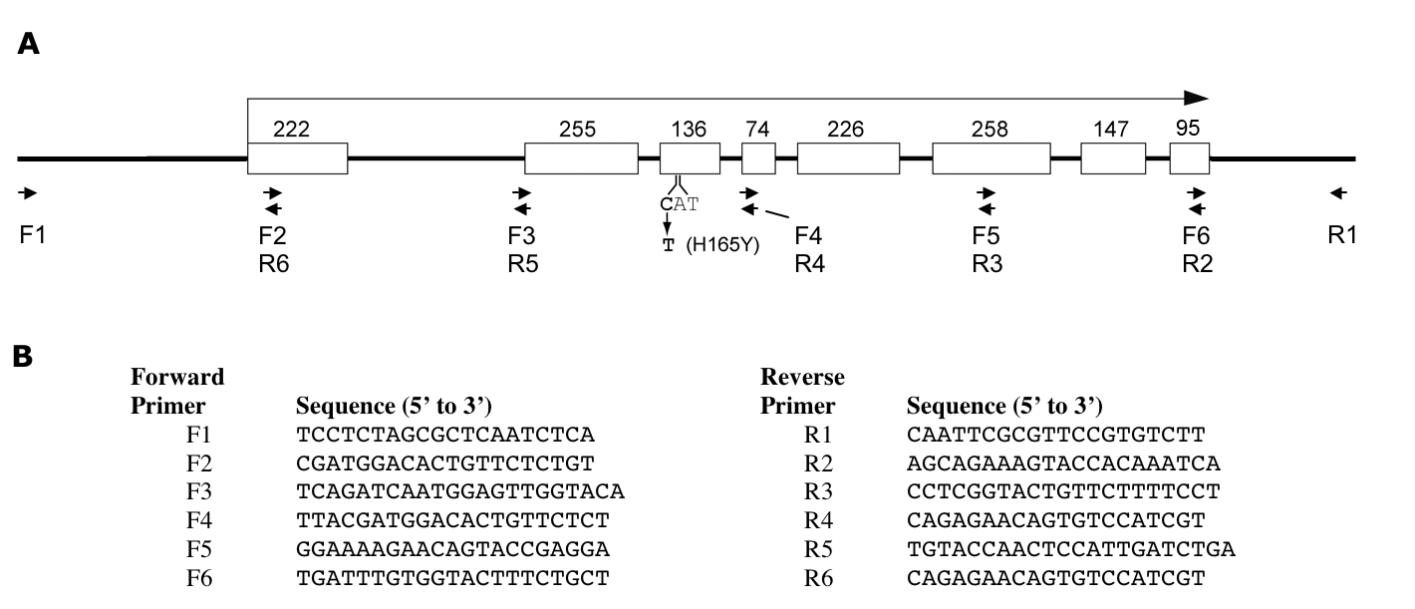
**Molecular analysis of *spe-4***. A. Structure of *spe-4*. The boxes represent exons with the number of base pairs above. The *hc196 *mutation is shown below the third exon. The arrows represent oligonucleotides used for PCR amplification and DNA sequencing. B. The sequence of the oligonucleotides.

**Figure 3:**
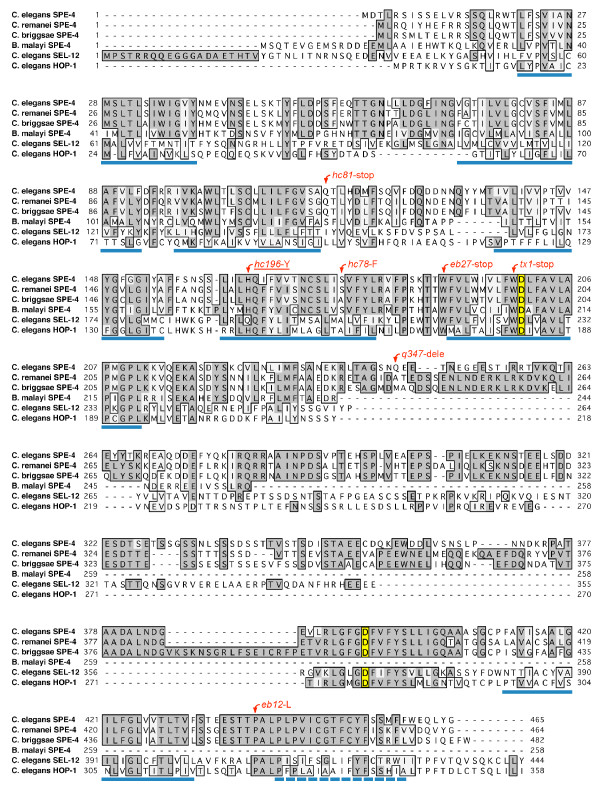
**Alignment of nematode SPE-4 protein sequences**. *C. elegans *SPE-4 is aligned with SPE-4 sequences from several nematode species and with *C. elegans *SEL-12 and HOP-1, the other presiniln orthologs in the *C. elegans *genome. Positions that are shaded and boxed represent identities, whereas boxed positions represent chemical similarity. The *hc196 *mutation is indicated above the altered amino acid, as are the other mutations in *C. elegans *SPE-4. The blue lines beneath the sequences represent transmembrane domains as predicted for *C. elegans *SPE-4 by TMPred (Release: TMBase25) [42]. The positions with yellow shading represent the catalytic aspartates involved in Presenilin 1 proteolysis as predicted by the ELM Server (Release: May 2007) [27]. Accession numbers for the protein sequences are: *C. elegans *SPE-4, *C. briggsae *SPE-4, CAE66611.1; *Brugia malayi *SPE-4, EDP33786.1; *C. elegans *SEL-12, NP_508175.1; and *C. elegans *HOP-1, NP_491328.1. The *C. remanei *SPE-4 sequence was obtained from WormBase.org, accession number RP:RP21351 (Release WS184).

Finally, we sequenced *spe-4 *from our *hc196 *strain and identified a missense mutation. Every base pair was covered by at least two sequencing reads from a cloned region of *hc196 *DNA containing *spe-4*. The mutation was confirmed by two additional sequencing reads on an independent PCR product amplified with primers F3 and R4 (Fig. 2). The mutation alters a histidine residue that is conserved in every nematode SPE-4 homolog known (Fig. 3). *spe-4 *is an ortholog of the human presenilin gene PS1, but a paralog two other PS1 orthologs in the *C. elegans *genome: *sel-12 *and *hop-1*. The SPE-4 amino acid position affected by the *hc196 *mutation is conserved in HOP-1 but not SEL-12. Our mutation lies in a membrane spanning domain, and the majority of the mutations isolated in SPE-4 (all of which are apparent null mutations) lie within 35 amino acids of *hc196 *(Fig. 3). Indeed, the *hc78 *missense mutation is nearby in the same transmembrane domain, suggesting that this domain is important to SPE-4 function, even though the catalytic aspartates involved in the proteolytic Presenilin-1 function are located downstream (Fig. 3).

In an attempt to uncover additional *spe-4 *suppressor alleles, we investigated a collection of *spe-27 *suppressor mutants isolated in the same mutagenesis that generated *spe-4(hc196) *[25]. Because *spe-4(hc196) *males produce very few cross progeny, we mated heterozygous males [*spe-4(hc196)/+; spe-27*] to hermaphrodites of the other suppressor strains (which were all in a *spe-27 unc-22 *background). The F1 were isolated and kept at 25°C. None of the additional alleles tested resulted in fertile F1 progeny (Table 3). The fact that all the additional alleles complement *spe-4(hc196) *indicates that none of the additional suppressor mutations tested were suppressor alleles of *spe-4*. As controls, we tested three *spe-6 *alleles that suppress the sterility of *spe-27 *[25], and all of them complemented *hc196 *as well. The absence of additional alleles is curious, given the fact that 18 suppressor alleles of *spe-6 *were isolated in the *spe-27 *suppression mutagenesis that produced *spe-4(hc196) *[20]. At 1398 base pairs, the coding sequence of *spe-4 *is a bigger target for mutation than that of *spe-6 *at 1140 base pairs, so we expected to find multiple *spe-4 *suppressor alleles. Perhaps *spe-6 *plays a more central role in the inhibitory pathway or *spe-4 *has fewer amino acids critical to the inhibitory pathway function.

**Table 3:** Results of complementation tests between *spe-4(hc196) *and additional *spe-27 *suppressor alleles.

**Suppressor Allele**	**Brood size ± SEM (N)**	**Results**
*hc166*	0.5 ± 0.2 (25)	Complementation
*hc176*	0.4 ± 0.2 (16)	Complementation
*hc197*	0.5 ± 0.1 (21)	Complementation
*hc198*	*	Complementation
*hc199*	0.1 ± 0.1 (15)	Complementation
*hc201*	0.1 ± 0.1 (8)	Complementation
*hc202*	*	Complementation
*hc204*	0.4 ± 0.3 (10)	Complementation
*hc205*	1.2 ± 0.6 (5)	Complementation
*zq6*	0 ± 0 (20)	Complementation
*zq7*	0.3 ± 0.2 (13)	Complementation
*zq8*	0.2 ± 0.1 (16)	Complementation
*spe-6(hc163)*	0.6 ± 0.2 (10)	Complementation
*spe-6(hc187)*	0.4 ± 0.2 (10)	Complementation
*spe-6(hc188)*	0.6 ± 0.2 (22)	Complementation

The brood sizes were measured in a *spe-27(it132ts) *background at 25°C.

* Complementation tests for these alleles were conducted by Paul Muhlrad [25]

### *spe-4(hc196) *is a recessive bypass suppressor of spermiogenesis pathway mutations and has a conditional Spe phenotype on its own

Worms homozygous for *spe-27(it132ts) *are sterile at 25°C, unless they are also homozygous for *spe-4(hc196) *(see Table 4). This suppression of *spe-27 *sterility is a recessive phenotype, as *spe-4(hc196)/+ *are sterile in a *spe-27 *background. In an otherwise wild-type background, *spe-4(hc196) *worms have reduced fertility that is influenced by temperature: they produce five times more progeny at 20°C than they do at 25°C (Table 4). This fertility deficit is a due to a sperm defect, because the fertility of *spe-4(hc196) *hermaphrodites increases after males supply sperm at mating (Table 4). Furthermore, the *spe-4(hc196) *phenotype is recessive; *spe-4(hc196)/+; spe-27/+ *worms have wild-type fertility (Table 4).

**Table 4:** Brood sizes of various strains. All *spe-27 *alleles were *it132ts*

	**Temperature-dependent Brood Size ± SEM (N)**		
	
**Hermaphrodite Genotype**	**15C**	**20C**	**25C**
*spe-4(hc196); spe-27 unc-22*	43.0 ± 7.2 (17)	64.0 ± 6.9 (21)	11.0 ± 0.7 (64)
*spe-4(hc196); spe-27*	59.8 ± 9.1 (13)	100.4 ± 8.6 (14)	14.6 ± 2.1 (14)
*spe-27 unc-22*	103.6 ± 11.7 (5)	4.2 ± 1.0 (4)	0.2 ± 0.2 (5)
*spe-27*	236.2 ± 17.2 (5)	2.0 ± 0.5 (5)	0.2 ± 0.1 (63)
*spe-4(hc196)/+; spe-27*			0.2 ± 0.1 (30)
*spe-4(hc196)/+; spe-27/+*			191.0 ± 19.0 (11)
*spe-27/+*			199.3 ± 11.8 (12)
*spe-4(hc196)*	84.8 ± 2.8 (5)	84.8 ± 4.8 (5)	16.6 ± 1.8 (27)
*spe-4(hc196) *× N2 males	135.2 ± 6.1 (4)		123.8 ± 15.3 (5)
*dpy-5(e61) spe-4(hc196); spe-8(hc134ts)*			18.6 ± 1.6 (9)
*spe-8(hc134ts)*			0.3 ± 0.2 (10)
*dpy-5(e61) spe-4(hc196); spe-19(eb52)*			15.2 ± 1.4 (19)
*spe-19(eb52)*			0 ± 0 (10)
*spe-4(hc196); spe-29(it129) dpy-20(e1282ts)*			14.2 ± 1.9 (20)
*spe-29(it129) dpy-20(e1282ts)*			0.1 ± 0.1 (10)
N2	303.6 ± 19.8 (5)	246.6 ± 12.2 (5)	190.8 ± 5.4 (5)

Comparing the *spe-4(hc196) *suppression phenotype with its own phenotype in a wild-type *spe-27 *genetic background suggests that *spe-4(hc196) *is epistatic to *spe-27*. The fertility of the double mutant more closely resembles *spe-4(hc196) *than it does *spe-27(it132ts) *(Table 4). This argument assumes that the suppression and sterile phenotypes are brought about by the same mechanism. If they are due to different mechanisms, then the epistatic relationship between these genes is not obvious from our data.

We were interested in the mechanism by which *spe-4(hc196) *suppresses *spe-27 *sterility. To test whether the interaction between the two genes is specific, we assessed the ability of *spe-4(hc196) *to suppress the sterility of mutations in the spermiogenesis genes *spe-8, spe-19*, and *spe-29 *(a double *spe-4 spe-12 *mutant was not made because *spe-12 *is tightly linked to *spe-4*; the two genes are only ~0.6 map units apart). In each case, *spe-4(hc196) *restored fertility to the spermiogenesis gene mutants (Table 4). Therefore, the suppression phenotype of *spe-4(hc196) *is not allele or gene specific, suggesting that it bypasses the spermiogenesis pathway in a manner similar to the *spe-6 *suppressor alleles described by Muhlrad and Ward [20].

We also tested three putative null *spe-4 *alleles for suppression of *spe-27*. These three alleles produce a sterile phenotype. One is a missense mutation (*hc78)*, while the other two are molecular nulls: *hc81 *(amber stop), and *q347 *(deletion) (Fig. 3). In each case, the *spe-4; spe-27 *double worms were sterile (Table 5). This result was not surprising, given that these *spe-4 *mutations cause spermatocyte arrest and the virtual absence of spermatids available to undergo spermiogenesis [22,23]. What was surprising was that the heterozygous state of each of the three alleles did suppress *spe-27 *(Table 5). The suppression of *spe-27 *by heterozygous *spe-4 *alleles that are null suggests that a simple reduction (but not elimination) of SPE-4 function is sufficient for suppression. It is interesting that suppression by *spe-4(hc78)/+ *resulted in larger broods than either of the two molecular nulls, suggesting that *spe-4(hc78) *is not a null allele, even though it induces complete sterility. Its residual function is only apparent in the suppression phenotype. Furthermore, the trans-heterozygote, *dpy-5 spe-4(hc196) +/+ spe-4(hc78) unc-15; spe-27 unc-22 *also suppressed *spe-27 *(Table 5), which is of interest because the trans-heterozygote was sterile when two both copies of the *spe-27 *gene were wild type (Table 1).

**Table 5:** Suppression of *spe-27 *by different *spe-4 *alleles.

**Hermaphrodite Genotype**	**Brood Size ± SEM (N)**
***spe-4(hc78)***	
*spe-4(hc78) unc-15*	0 ± 0 (32)
*spe-4(hc78) unc-15/*+ +	141.0 ± 11.8 (6)
*spe-4(hc78) unc-15; spe-27 unc-22*	0.7 ± 0.3 (9)
*spe-4(hc78) unc-15/++; spe-27 unc-22*	26.7 ± 3.6 (31)
***spe-4(q347)***	
*dpy-5(e61) spe-4(q347)*	0 ± 0 (9)
*dpy-5(e61) spe-4(q347)/+ +*	154.0 ± 25.0 (10)
*dpy-5(e61) spe-4(q347); spe-27 unc-22*	0 ± 0 (7)
*dpy-5(e61) spe-4(q347)/+ +; spe-27 unc-22*	13.8 ± 6.0 (14)
***spe-4(hc81)***	
*dpy-5(e61) spe-4(hc81)*	0 ± 0 (8)
*dpy-5(e61) spe-4(hc81)/+ +*	172.3 ± 33.3 (10)
*dpy-5(e61) spe-4(hc81); spe-27 unc-22*	0 ± 0 (8)
*dpy-5(e61) spe-4(hc81)/+ +; spe-27 unc-22*	10.4 ± 4.4 (11)
***spe-4(hc196)/spe-4(hc78)***	
*dpy-5 spe-4(hc196) +/+ spe-4(hc78) unc-15; spe-27 unc-22*	15.3 ± 3.8 (3)

Worms were reared at 25°C

### *spe-4(hc196) *causes multiple sperm defects including premature spermiogenesis

Mutant *spe-4(hc196) *hermaphrodites produce very few progeny at 25°C (Table 4). We mated these hermaphrodites to *fer-1(hc13ts); him-5 *males to see if more of their sperm would become functional after exposure to seminal fluid from *fer-1 *males, whose own sperm are defective. Such is the case with *spe-8 *group mutant hermaphrodite sperm, which activate and become functional when exposed to male seminal fluid [8,15,18]. We examined seven *spe-4(hc196) *hermaphrodites that had been paired with sterile *fer-1 *males: they produced an average of 151.1 progeny. Therefore, some of the spermatids within *spe-4(hc196) *hermaphrodites are similar to those in *spe-8 *group mutant hermaphrodites: they cannot activate unless exposed to male seminal fluid.

**Figure 4:**
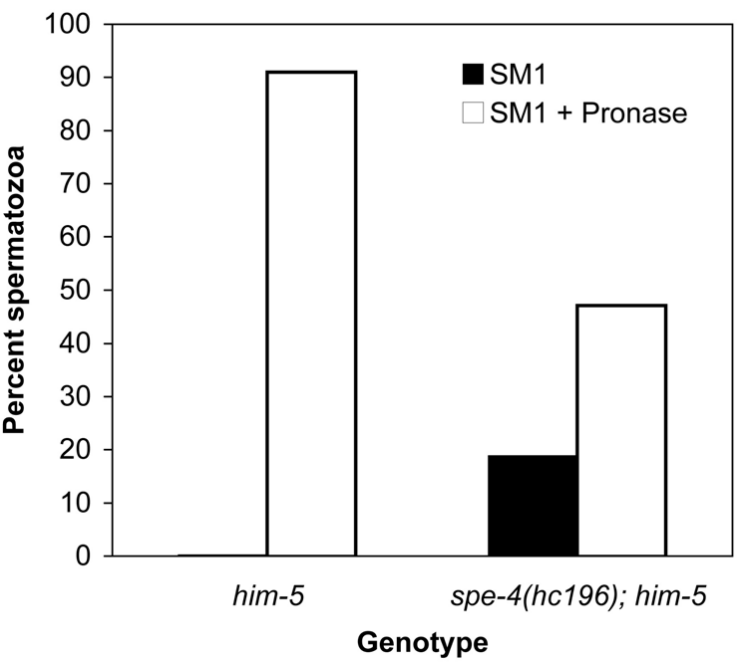
**Sperm activation in Pronase**. The presence of spermatozoa in sperm released from males dissected in sperm medium (SM1) and in sperm medium containing the protease Pronase, which activates spermatids to spermatozoa *in vitro*.

We also examined sperm from male *spe-4(hc196); him-5 *males. Compared to *him-5 *males, *spe-4(hc196); him-*5 male worms contained a large number of spermatocytes in the seminal vesicle where only spermatids should be found (CWL, personal observations), indicating that the *hc196 *mutation is an impediment to meiotic division, which is also a characteristic of the non-suppressor *spe-4 *mutations [22,23]. Some spermatocytes do successfully negotiate the meiotic divisions, and while mutant males accumulated spermatids, they also contained spermatozoa. In fact, nearly 20% of their sperm were crawling spermatozoa (Fig. 4). Typically, males store only spermatids, which undergo spermiogenesis after exposure to seminal fluid at ejaculation. The existence of spermatozoa within virgin males shows that they failed to pause in the spermatid stage. Furthermore, *spe-4(hc196) *males are nearly infertile, producing only small numbers of cross progeny even given 24 hours or more to mate. Such infertility may be due to premature spermatid activation because crawling spermatozoa impede sperm transfer during copulation [10], demonstrating the importance of the proper timing of sperm activation.

In contrast, not all the spermatids stored within *spe-4(hc196) *males appear capable of undergoing spermiogenesis. The ability of sperm to undergo spermiogenesis can be examined *in vitro *using Pronase, a collection of proteases which apparently cleave one or more cell surface components and thereby induce spermiogenesis [12]. While more than 90% of the spermatids in *him-5 *males activate to spermatozoa in the presence of Pronase, only ~50% of the sperm from *spe-4(hc196) *males activated in Pronase exposure (Fig. 4). Because 20% of the sperm these males contained was activated prior to exposure, Pronase treatment only activated 30% of the sperm. Therefore, almost 50% of the mutant sperm failed to activate. However, we did not observe any sperm that appeared "stalled" in the spiky intermediate stage of spermiogenesis. Remaining as spiky intermediates is a characteristic of the sperm from *spe-8 *group mutants when exposed to Pronase [8]. Thus, sperm from *spe-4(hc196) *mutants share some characteristics with the *spe-8 *group mutants that they suppress, but not all their characteristics.

**Figure 5:**
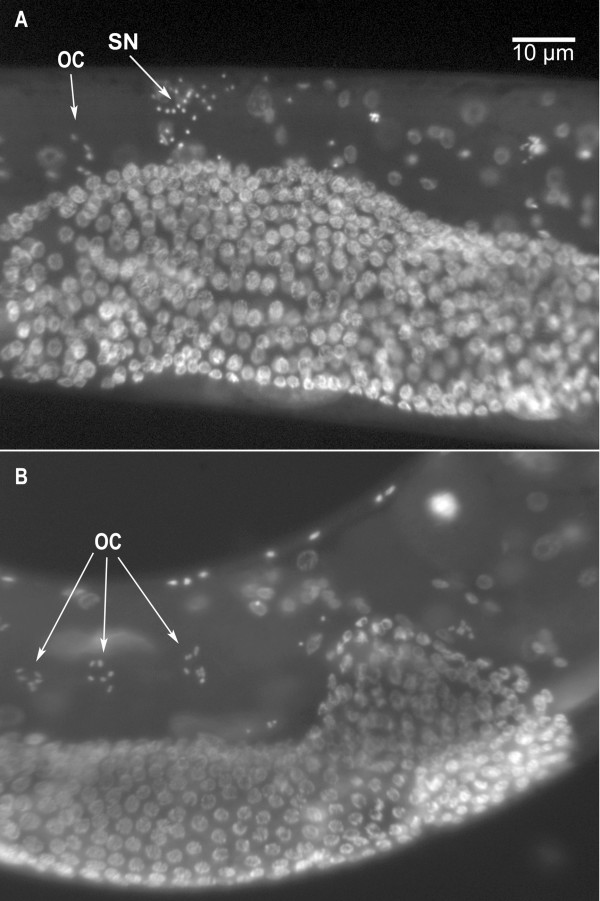
**Sperm from *spe-4(hc196) *males within *fer-1 *hermaphrodites**. Epifluorescence images of (A) a *fer-1(hc13ts) *hermaphrodite that had been mated to *spe-4(hc196); him-5(e1490) *males, and (B) an age-matched unmated *fer-1(hc13ts) *hermaphrodite showing the absence of sperm nuclei. SN indicates the characteristically compact DAPI labeled nuclei from the male's sperm [43], which appear localized to the spermatheca. OC indicates the oocyte chromosomes that are nearly the same size as sperm nuclei but occur in groups of 6 and are not as brightly fluorescent.

We had great difficulty obtaining cross progeny from *spe-4(hc196); him-5 *males, so we tested their fertility by pairing them with *fer-1(hc13ts) *hermaphrodites that had been raised at 25°C. The *fer-1 *mutation disrupts sperm function: *fer-1 *hermaphrodites are self-sterile and lose their defective self-sperm rapidly as they are swept from the reproductive tracts by passing unfertilized oocytes [26]. In 18 matings between individual *spe-4(hc196); him-5 *males each with four *fer-1 unc-29 *hermaphrodites, 13 males failed to sire any progeny, and together, the males averaged 1.22 progeny each (SD = 2.31). The males that sired no progeny actually did transfer sperm to the *fer-1 unc-29 *hermaphrodites, as evidenced by sperm within the mated hermaphrodites' spermathecae that were never found in unmated controls (Fig. 5). Therefore, at least some of the sperm from the mutant males were transferred during mating and crawled to the spermatheca, but they were unable to fertilize the oocytes.

### *spe-4 *is a member of an inhibitory pathway preventing premature spermiogenesis

**Figure 6:**
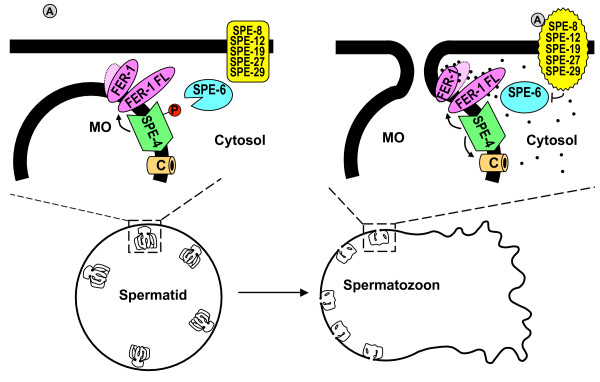
**Hypothetical model of SPE-4 function in spermatid activation**. In this model, SPE-6 phosphorylates downstream targets to maintain the spermatid stage [20]. We speculate that the Presenilin 1 homolog SPE-4 could be a target of SPE-6 kinase activity, increasing overall SPE-4 function. Based on the role of Presenilin 1 in cleaving amyloid precursor protein to produce two isoforms of amyloid β peptides, we further speculate that the potential proteolytic function of SPE-4 produces the two proteolytic isoforms of FER-1 found in sperm [14], although the target may be another protein. When the activation signal (A) is received and transduced by the *spe-8 *group proteins, SPE-6 function is suppressed, and SPE-4 function drops. There are at least two possible outcomes of reduced SPE-4 function: (i) a change in the ratio of the two proteolytic isoforms of FER-1 or another protein (denoted by the lighter and darker purple symbols for FER-1), which change in relation to spermiogenesis; and (ii) a spike in cytosolic Ca^2+ ^(black dots) mediated through SPE-4 itself or through a calcium channel (C). The result is the FER-1 mediated fusion of the MOs to the plasma membrane and pseudopod formation.

Because reduction of SPE-4 function bypasses the spermiogenesis activation pathway, *spe-4 *appears to be the second member of a spermiogenesis inhibition pathway. This pathway prevents progression through spermiogenesis until relieved by the signal transduced through the activation pathway consisting, at least in part, of *spe-8, spe-12, spe-19, spe-27*, and *spe-29 *(Fig. 6). The founding member of the inhibitory pathway is *spe-6*, a casein kinase I that is proposed to maintain inhibition through phosphorylation [20]. SPE-4 has multiple potential CK1 phosphorylation sites as predicted by the ELM Server (Release: May 2007) [27], so SPE-4 could be a direct target of SPE-6 CK1 activity.

The role of SPE-4 in the prevention of spermiogenesis may occur by several different mechanisms, but our results suggest that reduced SPE-4 function fosters precocious spermiogenesis. One possible mechanism involves the proteolytic function of the human PS1, a member of the γ-secretase complex whose members are located in the membranes of several cellular compartments including the Golgi. More than 150 mutations in PS1 contribute to early onset familial Alzheimer's Disease through abnormal deposition of the amyloid β-peptide (Aβ) in the brain [28,29]. Many of the PS1 mutations originally deemed to be gain of function are now thought to be loss of function lesions that decrease proteolytic efficiency but increase the ratio of the 42-residue to the 40-residue variant of Aβ [28,30]. We propose a model where the FER-1 protein is a target of SPE-4 proteolytic activity (Fig. 6). Spermatids contain two isoforms of FER-1, which likely result from proteolysis [14]. The human γ-secretase complex normally targets type I transmembrane proteins for proteolysis [31,32], but FER-1 is a type II membrane protein [33], casting doubt upon FER-1 as a target. However, we propose FER-1 as a SPE-4 target because (i) SPE-4 is the most divergent PS1 family member and may have different target specificities, (ii) both SPE-4 and FER-1 exist in the MO membrane, and (iii) FER-1 is cleaved during sperm maturation. In wild-type spermatids, activation likely happens when SPE-4 activity abates, perhaps as a result of decreasing SPE-6 function, resulting in an altered FER-1 isoform ratio that promotes spermiogenesis (Fig. 6). The hypothesis that FER-1 is a SPE-4 target requires further investigation.

Regulation of Ca^2+ ^homeostasis is another potential PS1-like function of SPE-4 that may play a role in the inhibition of spermiogenesis. PS1 mutations have been shown to perturb Ca^2+ ^homeostasis in human cells [34-36]. This perturbation may occur through a disruption in phosphatidylinositol 4,5-bisphosphate (PIP2) signaling [37]. PIP2 generally plays an important role in ion channel modulation [38]. Alternatively, SPE-4 might act as a Ca^2+ ^leak channel itself [39]. If SPE-4 regulates Ca^2+ ^homeostasis in spermatids either directly or through a PIP2-like intermediate, loss of function *spe-4 *mutations may affect sperm activation. SPE-4 resides in the MO membrane, the same location as TRP-3, a Ca^2+ ^channel (Fig. 6) [40]. It is not clear if TRP-3 functions in spermiogenesis, but intracellular chelation of Ca^2+ ^prevents spermiogenesis [14], indicating that spermiogenesis is Ca^2+ ^dependent. This leads to the hypothesis that SPE-4 maintains Ca^2+ ^sequestration in the spermatid phase and reduced SPE-4 activity allows Ca^2+ ^entry into the cytosol (Fig. 6). The exact role of SPE-4 in the inhibition of spermiogenesis remains to be determined.

The inhibitory pathway that consists of *spe-4 *and *spe-6 *likely contains multiple other genes and involves the membranous organelles. The *spe-27 *suppression screen that generated the suppressor alleles of *spe-4 *and *spe-6 *produced at least four other mutated genes, two and perhaps more of which are not previously known *spe *genes (unpublished data). Both SPE-4 and FER-1 reside in the MOs [14,22]. The MOs exist in a complex with the fibrous bodies to form the FB-MO complexes that function to package the major sperm proteins (MSPs). MSP packaging is deficient for the likely null alleles of both *spe-4 *and *spe-6 *[21,22]. The FBs disassemble in the spermatid as the MSP is released, but the MOs remain and fuse with the plasma membrane during spermiogenesis [8]. Therefore, the MO is an important site of activity during spermiogenesis, and it is conceivable that the machinery of the spermiogenesis inhibition pathway resides in the MOs.

It appears that the spermiogenesis inhibition pathway is important for normal sperm function. Males with the *spe-4(hc196) *mutation were largely sterile, even though mutant hermaphrodites were partly fertile. The male form of sterility likely derives from several sources. One is the fact that many mutant spermatids do not activate (if our *in vitro *activation assay reflects what occurs *in vivo*). Therefore, even though some spermatids activate prematurely, others apparently cannot activate at all. Another likely source of sterility is the obstruction that active spermatozoa pose to sperm transfer [10]. Finally some mutant sperm exhibit a fertilization defect. Perhaps these spermatozoa underwent spermiogenesis so prematurely that they were not properly organized for fertilization. This suggests that the timing of sperm activation is not only important for interaction with the external environment, but also for correct assembly of the spermatozoon. In our studies with *spe-4(hc196)*, premature activation results in only a small number of functional sperm; the majority are defective.

### SPE-4 sequence divergence

**Figure 7:**
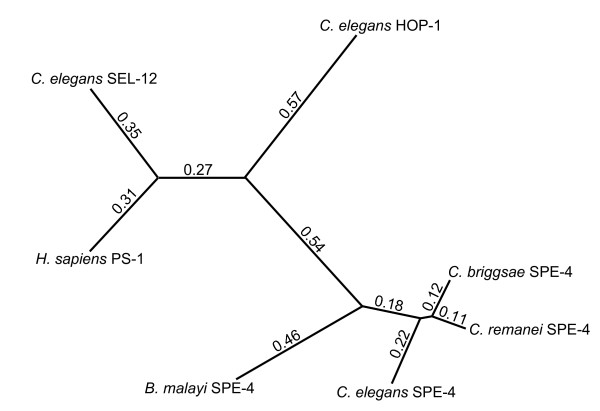
**Similarity relationships among selected nematode Presenilin-1 homologs**. The similarities among the protein sequences were calculated using a Neighbor-Joining method with Poisson correction. The numbers and branch lengths represent relative sequence differentiation among the sequences. In addition to the sequences found in Fig. 3, the human Presenilin 1 sequence accession number is BAD96893.1.

We performed an analysis of the similarities of the SPE-4 protein sequences from four nematodes along with those of SEL-12 and HOP-1, the two other *C. elegans *PS1 homologs, and human PS1 (Fig. 7). SEL-12 is the most similar of the nematode proteins to human PS1. HOP-1 is more diverged, and the four SPE-4 proteins cluster together. Therefore, it would appear that *hop-1 *and *spe-4 *are the result of duplication events since the separation of the human and nematode lineages. The *spe-4 *like genes are the most divergent of the PS1 orthologs, and because *Brugia malayi *has likely been separated from *Caenorhabditis spp. *for several hundred million years, the *spe-4 *type presenilins apparently have an ancient specialization that likely involves sperm function.

## Conclusion

*spe-4 *is the second member of a potentially new inhibitory pathway that prevents premature spermiogenesis in *C. elegans*. *spe-4(hc196) *was isolated in the same suppressor screen that produced multiple suppressor alleles of *spe-6*. This screen identified mutations that restored partial fertility to *spe-27(it132ts) *hermaphrodites. The suppressor alleles of both genes bypass the spermiogenesis signal transduction pathway to which *spe-27 *belongs, resulting in premature spermiogenesis. In addition to *spe-6 *and *spe-4*, the inhibitory pathway involves multiple other genes: we are investigating 4 other suppressor mutations, at least two of which do not map to known *spe *genes (unpublished).

Our results suggest that SPE-4 affects multiple aspects of sperm development. Earlier results showed that it aids in the packaging of MSP in primary spermatocytes [22,23]. We show that SPE-4 is also involved in spermiogenesis, and that one of the functions is inhibitory, preventing premature spermiogenesis until the spermiogenesis activation signal is received by the spermatid. The defects we observed in *spe-4(hc196) *mutants include (i) accumulation of excess spermatocytes, (ii) premature activation of some spermatids, but (iii) an inability of other spermatids to activate, although many defective hermaphrodite produced self-spermatids can be transactivated male seminal fluid. Finally some male sperm display a fertilization defect (iv), even though they navigate successfully to the spermatheca. This constellation of defects is difficult to explain. It is as if the *spe-4(hc196) *phenotypic trajectory for spermatids is binary. Either the spermatids fail to activate, or they activate prematurely resulting in some normal sperm and others that cannot fertilize. The fact that many mutant spermatozoa are incapable of fertilization suggests that the timing of spermiogenesis is very important. Premature spermiogenesis may preclude proper segregation of cellular components required for fertilization.

The *spe-4(hc196) *mutation described here should allow us to gain a better understanding of the cellular function of this presenilin homolog. It retains the most function of any *spe-4 *allele to be studied, and its newly-discovered role inhibiting spermiogenesis was unexpected, given its MSP packaging function early in spermatogenesis. The inhibitory role *spe-4 *plays in spermiogenesis may involve known proteolytic and/or calcium regulatory aspects of presenilin function, or it may involve yet-to-be discovered presenilin functions.

## Methods

### Worm strains and culture

All *C. elegans *strains were maintained on *Escherichia coli *OP50-seeded Nematode Growth Media (NGM) agar plates and manipulated as described by Brenner [41]. The *Caenorhabditis *Genetic Center kindly provided the following strains: N2, CB4856, *spe-27(it132ts)IV, spe-27(it132ts) unc-22(e66)IV, spe-29(it129) dpy-20(e1282ts)IV, him-5(e1490)V, spe-27(it132ts)IV; him-5(e1490)V, unc-22(e66)IV, dpy-5(e61)I; him-5(e1490)V, dpy-5(e61) unc-29(e193)I; spe-27(it132ts)IV, spe-8(hc53)I, spe-12(hc76)I, spe-19(hc41)V, fer-1(hc13ts)I, sDf5/spe-4(hc78) unc-15(e73)I*. We received the strain bearing the *hc196 *mutation, which was isolated in a suppressor screen of *spe-27(it132ts) *[20,25], from Samuel Ward at the University of Arizona. We backcrossed *hc196; spe-27(it132ts) unc-22(e66) *six times with *spe-27(it132ts)*, each time recovering *unc-22 *F2 that were fertile at 25°C. In addition to *hc196*, we also received numerous strains harboring mutations that suppress *spe-27(it132ts) *from Samuel Ward. The strains *sDf5/spe-4(q347) unc-13(e1091)I *and *sDf5/dpy-5(e61) spe-4(hc81)I *were kindly provided by Steven W. L'Hernault at Emory University.

### Single-nucleotide-polymorphism-based mapping of *hc196*

We mapped *hc196 *using its *spe-27(it132ts) *suppression phenotype. We constructed a mapping strain which had *spe-27(it132ts) *backcrossed into the Hawaiian strain CB4856 six times (henceforth HA-*spe-27*). The mapping cross consisted of *hc196; spe-27(it132ts) unc-22(e66) *hermaphrodites mated to HA-*spe-27 *males. Virgin F1 progeny were allowed to self-fertilize at 15°C, where *spe-27(it132ts) *worms are fertile. We isolated 237 F2 worms (as L4 larvae) and allowed them to self-fertilize at 25°C, where only *hc196 *homozygotes are fertile in a *spe-27 *background. Forty-five of the F2 were identified as fertile. These fertiles were combined for bulk DNA extraction, as were another 45 sterile F2 worms chosen arbitrarily, following the protocol of Wicks et al. [24]. We initially mapped *hc196 *to chromosome I using SNP loci on the following clones (Chromosome: nucleotide position): pKP1119 (I: 8,508,270), T13C2 (II: 6,789,140), B0280 (III: 7,130,643), AC3 (V: 10,365,871). We did not analyze SNPs on chromosome IV because preliminary data indicated *hc196 *was unlinked to *spe-27 *on chromosome IV. The *hc196 *interval on Chromosome I was mapped using SNPs on the following clones (clone: genetic map location: nucleotide position): pkP1101 (C54G6: -17.40: 992,188), pkP1106 (Y54E10A: -4.47: 3,163,067), pkP1109 (E01A2: -1.66: 4,132,728), pkP1055 (C09D4: 0.05: 5,482,534), snp_F26B1[1] (F26B1: 0.98: 6,324,724), pkP1117 (T23G11: 2.26: 7,686,868), pkP1119 (K02B12: 2.88: 8,508,270), and pkP1071 (C37A5: 23.41: 14,154,876). All SNP information based upon WormBase release WS187.

### Two- and three-factor mapping of *hc196*

To refine the SNP-mapped interval of *hc196*, we mapped the mutation in a *trans*-heterozygote cross with *dpy-*5. All strains had a *spe-27 *background, so that the *hc196 *phenotype was fertility. To further narrow the *hc196 *locus we performed a three-point mapping cross with *dpy-5(e61)I *(map locus 0.00) and *unc-29(e193)I *(map locus 3.32). These two genes were chosen because earlier map data suggested that they flank *hc196*. Male *hc196; spe-27 *males were mated to virgin *dpy-5 unc-29; spe-27 *hermaphrodites. Cross progeny (F1) were selfed at 15°C, and F2 recombinants (Dpy-non-Unc orUnc-non-Dpy) were isolated and raised at 15°C. F3 progeny from each recombinant F2 were checked for fertility at 25°C; those F3 that were fertile indicated that their F2 parent was heterozygous for *hc196*. The map locus was calculated based upon the number of recombination events between *dpy-5 *and *hc196*, and between *hc196 *and *unc-29*.

### Complementation crosses

To identify additional alleles of the gene mutated in *hc196*, we tested a number of other uncharacterized *spe-27 *suppressor mutations. Because *hc196 *males have only weak fertility, we used heterozygous males (*hc196/+; spe-27 unc-22/spe-27 +*) and crossed them with hermaphrodites of the strain to be tested, such as *hc197; spe-27 unc-22*. We isolated virgin, non-Unc F1 at 25°C and assessed fertility. If the two mutations were allelic, half of the F1s should be sterile and half should be fertile (noncomplementation). Alternatively, if they were different genes, all F1s should be sterile (complementation). In addition to *hc197*, the other *spe-27 *suppressor alleles tested included *hc166, hc176, hc199, hc201, hc204, hc205, zq6, zq7*, and *zq8*. These suppressor strains were all in a *spe-27 unc-22 *background. We performed control complementation tests with three *spe-6 *alleles that are known to suppress *spe-27 *sterility (*hc163, hc187*, and *hc188*). Based on the outcome of the genetic mapping, we also performed a complementation test with *spe-4*, which maps to 2.10 on chromosome I, very near the *hc196 *locus. *hc196*; *spe-27 *males were mated to *spe-4(hc78) unc-15 *hermaphrodites at 20°C. Virgin F1 were isolated at 25°C to assess fertility. Control crosses with N2 males mated to *spe-4 unc-15 *hermaphrodites were also performed.

### Molecular identification of *hc196 *as *spe-4*

We sequenced *spe-4 *from our *hc196 *worms using the Eppendorf TripleMaster PCR System™ for high fidelity PCR. The 2,852 bp fragment was amplified with primers F1 and R1 (Fig. 2) and extended from 451 bp upstream through 300 bp downstream of the *spe-4 *coding sequence. The PCR product was cloned using the AccepTor™ vector system and NovaBlue Singles™ competent cells (Novagen). Recombinant plasmids were purified from overnight cultures, and the insert was sequenced. In addition to the pETBlue up and pETBlue down vector primers, we constructed a set of sequencing primers dividing the insert into ~500 bp segments (Fig. 1). Automated DNA sequencing was performed by the Beckman Research Institute of the City of Hope (Duarte, CA) on a Hitachi AB model 3730 capillary DNA analyzer. The putative mutation identified by sequencing insert was verified by sequencing a PCR product amplified from *hc196 *genomic DNA containing the mutation.

### Microinjection transformation rescue

We amplified the same 2.8 kb PCR product described above from wild-type worms. Wild-type worms were microinjected with the *spe-4 *PCR product (20 ng/μl) along with *myo-2*::GFP (100 ng/μl) as a transformation marker. Transformed worms were cultured and the progeny examined for transformation. We recovered a strain of worms that transmitted the transgenes stably across generations (*zqEx1 [myo-2::GFP spe-4(+)]*). Transformed males were mated to *dpy-5 hc196; spe-27 unc-22 *hermaphrodites. F1 worms with GFP expression were isolated and F2 worms that were GFP positive and *dpy-5 *but not *unc-22 *were isolated and kept at 25°C. Their fertility was scored daily. As a control, wild-type males were used instead of transformant males.

### *spe-4 *suppression phenotype

We tested the ability of *spe-4(hc196) *to suppress *spe-8(hc53), spe-19(hc41)*, and *spe-29(it129)*, additional genes in the spermiogenesis activation pathway. To perform these tests, we constructed *spe-4(hc196) *double mutants with each of these genes and assessed their fertility at 25°C. We also tested the *spe-27 *suppression phenotype of three other *spe-4 *alleles that result in complete hermaphrodite sterility: *hc78*, *hc81*, and *q347*. Both *hc81 *and *q347 *are molecular null alleles, one being an amber stop and the other a nonsense deletion, respectively, while *hc78 *is a missense mutation that does not prevent production of SPE-4 protein [22]. For this assay, we crossed *spe-27 unc-22/spe-27+ *males with hermaphrodites of the three *spe-4 *lines. Each *spe-4 *line was marked with either an Unc or a Dpy morphological mutation (see Worm Strains section above for details). F1 hermaphrodites were selfed, and we recovered homozygous *spe-27 *F2 hermaphrodites (by picking *unc-22 *worms) of two classes : one class was homozygous for their particular *spe-4 *allele (homozygotes for the marker mutation), and the other class was not homozygous for the *spe-4 *allele (wild-type for the marker mutation). All F2 were reared at 25°C and assayed for fertility. Some of the F2 hermaphrodites that were not homozygous for *spe-4 *were fertile. In every case, they segregated either Unc or Dpy phenotypes in the F3, so they were *spe-4 *heterozygotes. Finally, *dpy-5 spe-4(hc196)/+ +; spe-27 unc-22/spe-27 + *males were mated into *spe-4(hc78) unc-15(e73) *hermaphrodites at 20°C. F1 were self-fertilized at 15°C and only *unc-22 *non-*dpy-5 *F2 were isolated at 25°C for fertility assay. These F2 were transheterozygotes for *spe-4 (dpy-5 spe-4(hc196) +/+ spe-4(hc78) unc-15; spe-27 unc-22*). The genotype was confirmed by observation of *dpy-5 *and *unc-15 *phenotypes in the F3.

## Authors' contributions

RG conducted most of the experimentation and contributed to manuscript preparation. WSL participated in the single nucleotide polymorphism mapping and transformation rescue experiments. CWL contributed to the microscopy and manuscript preparation. All authors read and approved the final manuscript.
